# Cardiovascular diseases health literacy among Italian navy personnel: A cross-sectional survey

**DOI:** 10.1016/j.pmedr.2025.102978

**Published:** 2025-01-18

**Authors:** L. Rondinini, V. Di Fiore, M. Pasquale, G. Cruschelli, D. Rocchi, F. Baglivo, A. Baggiani, A.D. Porretta

**Affiliations:** a Italian Navy Medical Service, Livorno, Italy; b Department of Translational Research and New Technologies in Medicine and Surgery, University of Pisa, Pisa, Italy

**Keywords:** Cardiovascular diseases, Cardiovascular risk factors, Prevention, Awareness, Navy, Cross-sectional survey

## Abstract

**Objectives:**

This study assesses cardiovascular disease (CVD) awareness among Italian Navy personnel, emphasizing the understanding of risk factors. It aims to provide insights that could enhance health education initiatives tailored to military settings.

**Study Design:**

Cross-sectional survey.

**Methods:**

From February 2021 to March 2022, participants were enrolled through opportunistic sampling among Italian Navy personnel. They were surveyed using a structured questionnaire divided into two parts. It was designed to elicit open-ended and closed-ended responses on their knowledge of CVD, its risk factors, and associated diagnostic thresholds. Statistical analyses included logistic regression conducted with R software.

**Results:**

A total of 785 participants were enrolled. The findings indicate a higher recognition of acute myocardial infarction and hypertension compared to other diseases like atherosclerosis and cardiomyopathy. For risk factors, smoking was recognized by 57 % of respondents, while only 60 % correctly identified the diagnostic threshold for total cholesterol. Logistic regression analysis showed significant differences in awareness levels based on sex and rank (*p* < 000000.1); officers had higher awareness than enlisted personnel (p < 000000.1).

**Conclusions:**

The results demonstrate significant disparities in CVD risk awareness among different ranks and sexes within the Italian Navy, highlighting the need for tailored educational programs. Through specific interventions, Addressing these knowledge gaps could enhance the health outcomes of military personnel and the overall efficacy of Navy health strategies. Furthermore, the work and life environment of the Navy could allow for more in-depth health education initiatives, leading to the adoption of healthier lifestyles by the personnel.

## Highlights

• This study assessed cardiovascular disease risk factors knowledge among 785 Italian Navy personnel.
• Noticeable gaps were identified in cardiovascular disease risk factor awareness across military ranks.
• The study showed how gender and rank influence health literacy within the military.
• Officers demonstrated higher levels of cardiovascular disease awareness than enlisted personnel
• Tailored health education initiatives are essential among military personnel.

## 1 Introduction

Cardiovascular diseases (CVD) are among the leading causes of death worldwide. In Europe, CVD are responsible for over half of all deaths, with an estimated 17.9 million deaths annually, of whom coronary artery disease and stroke are the primary culprits (Organization WH, 2024; Mach et al., 2020).

Given the importance of CVD for public health, it is essential to be aware of the risk factors to take adequate preventive or corrective measures. Cardiovascular risk awareness refers to understanding and recognizing factors that increase the risk of developing CVD (Kavey et al., 2015; Kotseva et al., 2009), such as high blood pressure, high cholesterol, obesity, smoking, and a sedentary lifestyle. The importance of CVD risk awareness is even more pronounced among military personnel, such as the Italian Navy, with their peculiar stressors and lifestyle factors that could potentially elevate CVD risks.

In the literature, no studies specifically focused on personnel from the Italian Navy. However, various international studies on military personnel and their health status (Belding et al., 2022; Xie et al., 2018) indicate that, while active service members generally enjoy good health, thanks in part to their active lifestyle and access to dedicated healthcare services, the continuous exposure to stressors and the unique demands of their work—often involving deployment in challenging environments for extended periods—places them at higher risk for factors such as sleep deprivation, smoking, poor diet, and alcohol consumption (McCabe et al., 2018a). This, in turn, contributes to an elevated cardiovascular risk. Indeed, some studies show that American veterans, upon completing their service (Golenbock et al., 2017; Hoerster et al., 2012), generally experience poorer health compared to civilians, including in terms of cardiovascular health.

In light of this, assessing cardiovascular risk awareness among active-duty military personnel is crucial, as it could inform targeted awareness campaigns and promote healthy lifestyle behaviors explicitly tailored to this population.

Despite media efforts to raise awareness of healthy lifestyles, there needs to be CVD risk awareness, particularly among young adults (Bucholz et al., 2018). This can be a worrying fact, especially for diseases like atherosclerosis that start early in life. Addressing this gap is crucial, as up to 80 % of CVD could be prevented by eliminating health risk behaviors (Sidney et al., 2016; Sulo et al., 2014).

### 1.1 Objective

The primary objective of this study is to assess CVD risk awareness among a sample of Italian Navy personnel, a population with a unique opportunity due to its relatively young age and supposedly healthy status. In this way, it will be possible to determine the knowledge level of the key factors contributing to cardiovascular health, as outlined by major international scientific organizations.

A secondary objective is to determine, among population study, the level of knowledge and the threshold of traditional cardiovascular risk factors, a topic rarely studied in the existing literature (Sarpong and Williams, 2024; Roever et al., 2018).

By evaluating the CVD risk awareness and the comprehension of critical thresholds, this study aims to provide insights into cardiovascular health literacy within this military cohort that could lead to more effective health education initiatives and prevention strategies tailored to military settings.

## 2 Methods

### 2.1 Participant recruitment and selection criteria

A questionnaire about CVD, related risk factors, and threshold for diagnostic criteria was administered. Participants in the cross-sectional study were enrolled by opportunistic sampling among Italian Navy personnel. During the mandatory biennial medical suitability examination, recruitment occurred from February 2021 to March 2022 at two designated sites: the Naval Academy in Livorno (Italy) and the Naval Medical Centre in La Spezia (Italy).

To ensure the anonymity of responses and to prevent duplicate submissions, a systematic record-keeping process was implemented, utilizing non-identifiable participant codes.

The inclusion criteria were active service members aged between 18 and 60 years with no diagnosed CVDs. This condition was verified through the medical screenings that are a part of the routine health assessment of naval personnel.

For the purposes of this study, biological sex was recorded, as it is a recognized factor in cardiovascular risk.

### 2.2 Questionnaire design and structure

The questionnaire developed for the study was divided into two distinct parts, administered sequentially to reduce potential response bias, with instructions to complete Part A before answering Part B.

The questionnaire was developed to match the recognized modifiable risk factors identified in the Framingham Risk Score, which includes smoking, arterial hypertension, LDL cholesterol, diabetes mellitus, obesity, and a sedentary lifestyle (D'Agostino et al., 2013).

The face validity of the questionnaire was assessed by a group of experts to ensure its relevance and appropriateness for evaluating CVD awareness in this military population.

#### 2.2.1 Part a: Open-ended knowledge assessment

This section of the questionnaire collected demographic information from participants, excluding any identifying data to preserve anonymity. It included an assessment scale of participants' self-perceived knowledge of CVD risk factors on a scale of 1 to 5, ranging from very low to high. In addition, two open-ended questions were included, inviting respondents to list all the CVD risk factors and diseases they were aware of. Responses utilizing non-technical language were systematically interpreted and categorized by the research team into their corresponding medical terminologies (e.g., “high levels of fat in blood” was classified as “dyslipidemia”).

#### 2.2.2 Part B: Close-ended knowledge assessment

The next phase of the questionnaire included 28 closed-ended questions designed to quantitatively assess the participants' knowledge regarding CVD, their risk factors, and specific threshold levels for blood pressure and cholesterol. The questionnaire was divided into three subscales: seven items focused on CVD, twelve on cardiovascular risk factors, and the remaining assessed knowledge of blood pressure and cholesterol thresholds. Response options for these items were standardized as “yes”, “no”, or “I don't know”.

### 2.3 Statistical analysis

Each participant's response form was archived, and the data were transcribed into an electronic database for subsequent statistical examinations.

The scoring mechanism involved calculating different scores for the open-ended and closed-ended responses. The open-ended scores for CVD and risk awareness were based on the number of diseases or risk factors correctly identified by respondents in part A. Similarly, the closed-ended scores were based on the number of correct answers in part B.

The Shapiro-Wilk normality test was applied to evaluate the distribution of open-ended and closed-ended responses.

In the case of non-normality, Spearman's rank correlation test was utilized to investigate the relationship between open-ended and closed-ended responses related to awareness of CVD and their associated risk factors.

Logistic regression analysis was performed using a combined awareness score. First, univariate analyses were performed to explore correlations between the combined awareness scores and demographic variables, such as gender, age, education level, and rank. Then, a multivariate regression analysis, focusing on gender and rank as independent variables, was used to verify critical predictors of CVD awareness.

A *p*-value of 0.05 or less was considered indicative of statistical significance. All statistical analyses were carried out using R software version 4.2.2.

### 2.4 Ethical considerations

The study was conducted following the Helsinki Declaration. It was authorized and approved by the Maritime Military Medical Corps as part of the comprehensive fitness assessment for military personnel's service as stated in the Directive for medical readiness visits (SMM SAN 001, 2023).

## 3 Results

### 3.1 Study population description

**Fig. 1:**
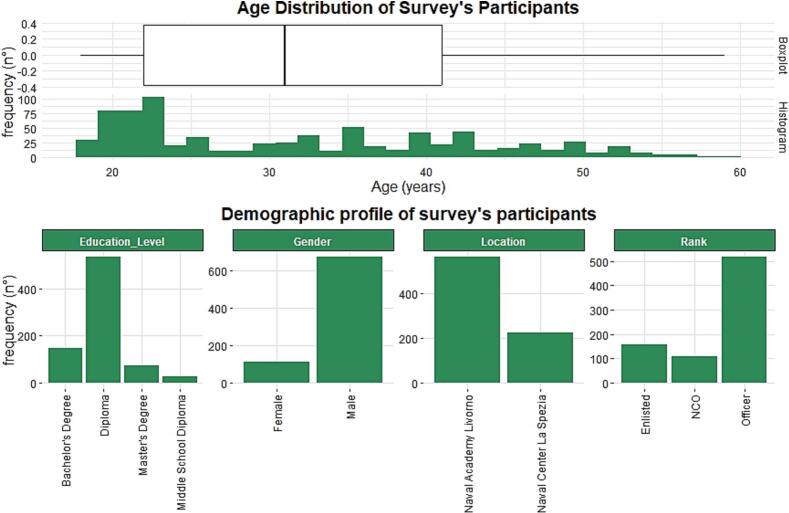
Sociodemographic characteristics of the enrolled navy personnel (Italy 2021–2022).

The participant sample included *n* = 785 participants recruited in two locations: the Naval Academy in Livorno (*n* = 560 participants, 71.3 %) and the Naval Medical Centre in La Spezia (*n* = 225 participants, 28.7 %). Most participants were male (*n* = 672, 85.6 %), as shown in Fig. 1. The sample was predominantly young, with a modal age of 20 (*n* = 80 participants). The mean age was 31.9 years (IQR 22–41, minimum 18, maximum 59), as shown in Fig. 1.

As regards the rank within the Italian Navy, the largest group was Officers (*n* = 517 participants, 65.8 %), followed by Enlisted (*n* = 158 participants, 20.1 %) and Non-commissioned officers (NCO) (*n* = 110 participants, 14.1 %).

The educational background of the participants is different, with the majority of them having a high school diploma (*n* = 535 participants, 68.1 %), followed by those with a Bachelor's Degree (*n* = 149 participants, 18.9 %), a Master's Degree (*n* = 73 participants, 9.3 %), and a Middle School Diploma (*n* = 28 participants, 3.7 %).

The results of Part A and Part B of the questionnaire were stratified for each socio-demographic variable. A statistical analysis was conducted to identify possible correlations between the variables before stratification, particularly rank, age, and education level.

An analysis of variance (ANOVA) was used to examine the relationship between rank and age. The results of the ANOVA test showed a significant difference in the mean age between different ranks (F-value = 288.9, *p*-value <0.00001), concluding that age differs significantly with rank.

A Chi-Square test of independence was used to assess the association between rank and education level. The test yielded a Chi-Square statistic of 120.21 with a p-value <0.00001, suggesting a highly significant association between these variables.

Higher ranks are associated with both higher education levels and older age.

### 3.2 Normality test results

The Shapiro-Wilk normality test was applied to the CVD awareness scores. Specifically, the open-ended and closed-ended questions had W statistics of 0.93 and 0.87, respectively, with *p*-values <0.00000001.

Similarly, questions on awareness of CVD risk factors showed W statistics of 0.96 and 0.92, respectively, with p-values <0.00000001. The test results indicated that none of these scores conformed to a normal distribution, indicating a significant deviation from a normal distribution for all types of awareness scores.

### 3.3 Results of part a: Open-ended knowledge assessment

The results from Part A of the questionnaire were stratified by demographic characteristics, which is essential for understanding the nuances in awareness levels of CVD, as these factors are likely to influence knowledge and perception of health risks.

The results indicate that **acute myocardial infarction** is the most recognized CVD among the different ranks, with 50.7 % of Officers, 55.5 % of NCOs, and 41.1 % of Enlisted. **Arterial hypertension** is the second most recognized condition, with 36.2 % of Officers, 45.5 % of NCOs, and 27.8 % of Enlisted being aware of it. **Stroke and thrombosis** were also recognized to a notable degree, with stroke being identified by 23.4 % of Officers, 15.5 % of NCOs, and 25.9 % of Enlisted, and thrombosis by 14.5 % of Officers, 6.4 % of NCOs, and 5.1 % of Enlisted. Lesser-known diseases included **atherosclerosis, valvular heart disease, myocarditis, cardiomyopathy, and aneurysm**, each recognized by less than 10 % of participants across all ranks. A complete report of the identified CVD is available in Supplementary Table 2.

Among the risk factors for CVD identified by participants in the Part A questionnaire, smoking is the most recognized, with 57.6 % of Officers, 66.4 % of NCOs, and 57.6 % of Enlisted acknowledging it. The second most recognized risk factor is a sedentary lifestyle, identified by 37.9 % of Officers, 45.5 % of NCOs, and 36.1 % of Enlisted. Obesity is identified by 34.2 % of Officers, 32.7 % of NCOs, and 25.9 % of Enlisted. A complete report of the risk factors identified is available in Supplementary Table 3.

#### 3.3.1 Part a scores

A score was calculated for awareness of CVD and their risk factors, giving 1 point for each correct disease or risk factor identified in part A of the questionnaire.

For open-ended questions on awareness of CVD and related risk factors, the mean scores were 1.60 (SD 1.79) and 2.61 (SD 1.79), respectively.

**Fig. 2:**
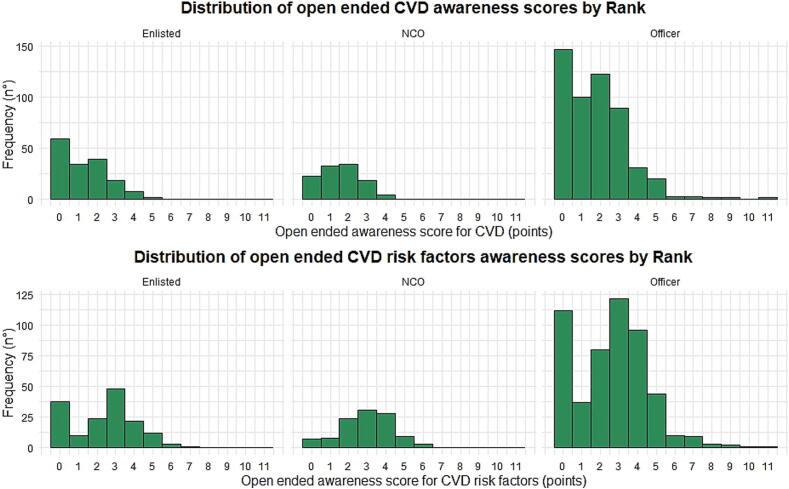
Results of open ended questionnaire on cardiovascular diseases health literacy among navy personnel (Italy 2021–2022).

Results stratified by rank are available in Supplementary Table 4, Supplementary Table 5, and Fig. 2.

### 3.4 Results of part B: Close-ended knowledge assessment

**Fig. 3:**
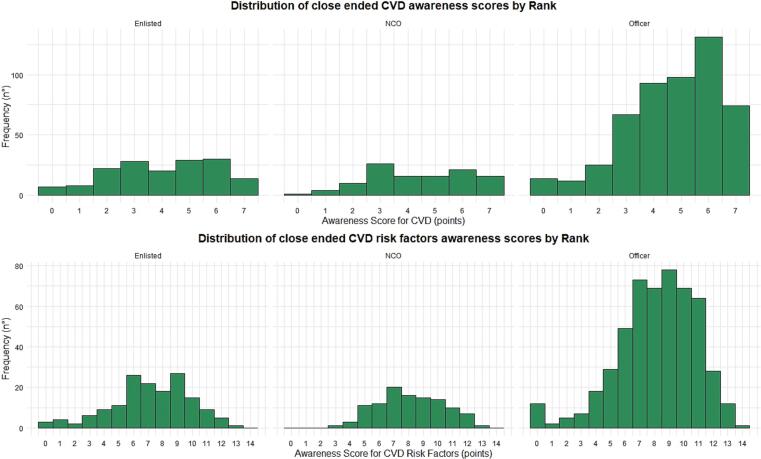
Results of close ended questionnaire on cardiovascular diseases health literacy among navy personnel (Italy 2021–2022).

A score was calculated for the assessment of knowledge of CVD (first section of the Part B questionnaire), with 1 point for each question answered correctly and 0 points for each question answered incorrectly or not at all, up to a maximum of 7 points. The mean score was 4.54/7 (SD 1.78). Results stratified by rank are available in Supplementary Table 7 and Fig. 3.

For the second section of the Part B questionnaire, a score was calculated with 1 point for each correct question and 0 points for each question answered incorrectly or not at all, up to a maximum of 14 points. The mean overall score was 7.955 (SD 2.660), with results stratified by rank shown in Supplementary Table 8 and Fig. 3.

### 3.5 Correlation test results

Spearman's rank correlation test was used to examine the relationship between scores from closed and open-ended questions, as the data did not follow a normal distribution. The test revealed a positive but weak correlation between the CVD awareness questions, with a correlation coefficient (rho) of 0.31 and a *p*-value <0.0000001. A similar pattern was observed between the CVD risk factors awareness questions, where the correlation coefficient was 0.29 with a p-value <0.0000001, again confirming a statistically significant but weak positive correlation.

### 3.6 Logistical regression analysis

**Fig. 4:**
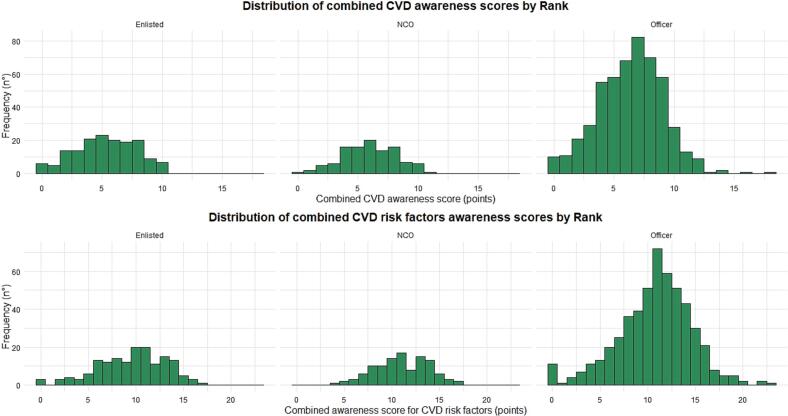
Total scores of the questionnaire on cardiovascular disease health literacy among navy personnel (Italy 2021–2022).

A combined awareness score for the logistical regression has been calculated for CVD and CVD risk factors by adding open-ended and close-ended scores. The mean combined awareness score for CVD was 6.13 (SD 1.79). The mean combined awareness score for CVD risk factors was 10.56 (SD 1.79). Results stratified by rank are available in Supplementary Table 9, Table 10, and Fig. 4.

Univariate analyses were conducted for gender, age, education level, and rank on the combined CVD and CVD risk factors awareness. Only gender and rank were found to be statistically significant. Therefore, a multivariate regression was performed with gender and rank as independent variables. Full results are available in Supplementary Table 11.

The result of the multivariate regression analysis with combined CVD awareness as the dependent variable shows that being male is statistically associated (*p* < 0.05) with a lower combined CVD awareness score than females. NCOs have a higher combined CVD awareness score compared to Enlisted. Officers also have a significantly higher combined CVD awareness score compared to Enlisted.

The multivariate regression analysis on the Navy dataset, with combined CVD risk factors as the dependent variable, revealed a negative association between being male and the combined CVD risk factors awareness score compared to females. It also suggests that NCOs have a higher combined CVD risk factor awareness score than the Enlisted and that Officers have a significantly higher combined CVD risk factor awareness score than the Enlisted.

The results suggest that gender and rank are important predictors of combined CVD and CVD risk factors awareness scores among Italian Navy personnel.

## 4 Discussion

The results of our study reveal notable gaps in CVD awareness among Italian Navy personnel, with certain conditions, such as myocardial infarction and hypertension, more frequently recognized, while others, including atherosclerosis and cardiomyopathy, remain less understood. This highlights the importance of targeted health literacy initiatives to address these gaps and improve overall cardiovascular awareness within the military context, as suggested by previous studies based on military personnel in other countries (McCabe et al., 2018b; Haibach et al., 2017).

Specifically, the results indicate that CVDs more frequently recognized are acute myocardial infarction, arterial hypertension, stroke, and thrombosis. At the same time, other important conditions, such as atherosclerosis, valvular heart disease, myocarditis, cardiomyopathy, and aneurysm, are less recognized.

Our sample can be considered representative of the Italian Navy, as it reflects the distributions of biological sex and age. According to the 2023 report on the Italian Navy, there are approximately 38,500 personnel, with an average age of 41 years. Around 90 % of personnel are male in terms of biological sex distribution. Rank distribution is approximately 15 % officers, 43 % NCOs, and 42 % enlisted personnel. The one exception in representativeness is in rank distribution, as the presence of the Officers' Academy in Livorno, one of our survey locations, resulted in a higher proportion of responses from officers.

The same observation can be made for risk factors, where smoking, a sedentary lifestyle, and obesity are among the most known, together with atherosclerosis. For instance, diabetes mellitus is recognized as such by less than 15 % of the participants and by less than 50 % in the close-ended questions.

Moreover, only 60 % of the participants correctly identified the cut-off value for total cholesterol. Similarly, the responses show that less than 50 % of the participants correctly identified the threshold for arterial pressure.

The positive but weak correlation between open-ended and closed-ended questionnaire responses suggests that while participants are generally aware of CVD and their risk factors, their knowledge needs more depth and precision. This finding aligns with previous studies that have described similar trends in young adults and military populations, where understanding cardiovascular risks often does not translate into accurate knowledge of diagnostic criteria or less common risk factors  (Trejo et al., 2018).

The observed differences in awareness according to gender and rank are also noteworthy. Females and Officers demonstrated higher levels of cardiovascular awareness compared to their counterparts. This disparity could be due to variations in education, health literacy, and perhaps differing health promotion efforts targeted at different ranks and genders within the military. These findings suggest that tailored educational initiatives considering these demographic differences could raise awareness across the board more effectively.

Despite CVD being the leading cause of both morbidity and mortality, some diseases, such as coronary artery disease, have shown a halving since the 1980s. Preventive measures, such as the sensibilization campaign against cigarette smoking – also at the legislation level – have been promoted in Europe despite inequalities between countries. However, many risk factors have increased in prevalence^13,^ primarily obesity and diabetes mellitus.

Health education and promotion are practical tools for the prevention of CVD. Education campaigns and public health initiatives to promote healthy lifestyles can increase awareness of the risk factors for CVD and encourage people to adopt healthy behaviors.

Cardiovascular prevention has achieved significant objectives in the past decades, as it was recently reported that the increase in CVD mortality in younger adults has slowed (Bucholz et al., 2018) or has been absent (Sidney et al., 2016; Piepoli et al., 2016). That is even more correct in young adults, whose knowledge of cardiovascular risk factors is understudied, generally resulting lower than adults (Facts, 2013; Lynch et al., 2006), to prevent clinical manifestations of atherosclerosis that, as well known, begin early in life.

In addition, a limitation of this study is the lack of weight-related health data, which could further inform cardiovascular risk assessment; however, as active military personnel, participants are generally required to meet specific physical standards, including weight criteria, which may mitigate this factor.

The effectiveness of cardiovascular risk awareness campaigns can be challenging to measure, as the impact of these campaigns may be soon apparent. However, studies have shown that these campaigns can effectively increase knowledge and improve behaviors related to heart health (i.e., SMART Project, Bogalusa Heart Study) (Güneş et al., 2019).

## 5 Conclusions

This study shows that awareness of CVD and related risk factors could be improved in specific demographic groups. This could reduce the population's overall burden of heart disease and improve the overall health of individuals and communities. Increasing awareness of cardiovascular risk can also help identify people who are at high risk of developing CVD and who may benefit from early intervention and treatment. Because of the lack of knowledge about these risks, developing awareness campaigns to educate people about them would be beneficial. Identifying categories with lower levels of awareness is important for planning future interventions, as most educational and training activities within the Italian Navy are addressed to specific groups of the same rank and/or role. It is possible to create content for the Health Education and Promotion initiative tailored to the particular needs identified in this survey and the level of formal education of the single target group, as defined by their rank. The observed coherence between open-ended and closed-ended questions validates the questionnaire as the basis for future surveys, especially for pre- and post-educational initiatives to evaluate their effectiveness.

In conclusion, enhancing CVD awareness among Italian Naval personnel is crucial for reducing the risks associated with CVD. Tailored health education campaigns that address identified knowledge gaps and take advantage of demographic insights can promote a more informed and health-conscious military population, ultimately contributing to better cardiovascular health outcomes and operational readiness.

## Funding

This research received no specific grant from funding agencies in the public, commercial, or not-for-profit sectors.

## Declaration of generative AI and AI-assisted technologies in the writing process

During the preparation of this work, the author FB used ChatGPT (GPT-4o) and Grammarly in order to improve the language and clarity of the draft. After using this tool/service, all the authors reviewed and edited the content as needed and take full responsibility for the content of the publication.

## Contributors

LR and VDF contributed to the conception and design of the study. FB contributed to the statistical analysis. FB, GC, VDF, MP, and DR contributed to writing the manuscript. ADP and LR contributed to the critical revision of the manuscript, and all authors approved the manuscript.

## CRediT authorship contribution statement

**L. Rondinini:** Writing – review & editing, Supervision, Conceptualization. **V. Di Fiore:** Writing – review & editing, Supervision, Conceptualization. **M. Pasquale:** Writing – original draft. **G. Cruschelli:** Writing – original draft. **D. Rocchi:** Writing – original draft. **F. Baglivo:** Writing – original draft, Visualization, Methodology, Formal analysis, Data curation. **A. Baggiani:** Writing – review & editing, Supervision, Funding acquisition. **A.D. Porretta:** Writing – review & editing, Supervision, Methodology, Funding acquisition, Conceptualization.

## Declaration of competing interest

The authors declare that they have no known competing financial interests or personal relationships that could have appeared to influence the work reported in this paper.

## Data Availability

The authors do not have permission to share data.

## Appendix A Supplementary data

Supplementary material: **Supplementary Table 1** - Demographic and Educational features of the respondents. **Supplementary Table 2** - Answers of CVD reported in Part A. **Supplementary Table 3** - Answers of CVD risk factors reported in Part A. **Supplementary Table 4** - Results of answers about open-ended CVD stratified by rank in Part A. **Supplementary Table 5** - Results of answers about open-ended CVD risk factors stratified by rank in Part A. **Supplementary Table 6** - Results of Part B: Close-ended knowledge assessment. **Supplementary Table 7** - Results of answers about close-ended CVD stratified by rank in Part B. **Supplementary Table 8** - Results of answers about close-ended CVD risk factors stratified by rank in Part B. **Supplementary Table 9** - Results of mean combined awareness score for CVD. **Supplementary Table 10** - Results of mean combined awareness score for CVD risk factors. **Supplementary Table 11** - Multivariate regression was performed with gender and rank as independent variables. Image 1
