# Supplementary material for: Cardiovascular diseases health literacy among Italian navy personnel: A cross-sectional survey
Source: Prev Med Rep. 2025 Jan 18;50:102978. doi: 10.1016/j.pmedr.2025.102978 (PMC11795100; doi:10.1016/j.pmedr.2025.102978)
Supplement: Supplementary file 1 — Supplementary material: Supplementary Table 1 - Demographic and Educational features of the respondents. Supplementary Table 2 - Answers of CVD reported in Part A. Supplementary Table 3 - Answers of CVD risk factors reported in Part A. Supplementary Table 4 - Results of answers about open-ended CVD stratified by rank in Part A. Supplementary Table 5 - Results of answers about open-ended CVD risk factors stratified by rank in Part A. Supplementary Table 6 - Results of Part B: Close-ended knowledge assessment. Supplementary Table 7 - Results of answers about close-ended CVD stratified by rank in Part B. Supplementary Table 8 - Results of answers about close-ended CVD risk factors stratified by rank in Part B. Supplementary Table 9 - Results of mean combined awareness score for CVD. Supplementary Table 10 - Results of mean combined awareness score for CVD risk factors. Supplementary Table 11 - Multivariate regression was performed with gender and rank as independent variables. [file mmc1.docx]

Supplementary Materials

[SUPPLEMENTARY Table 1 - Demographic and Educational features of the respondents.](#_5hi2nuak6860) 1

[SUPPLEMENTARY Table 2 - Answers of CVD reported in Part A.](#_lnxisoik93zm) 3

[SUPPLEMENTARY Table 3 - Answers of CVD risk factors reported in Part A.](#_moc17yvk3gac) 4

[SUPPLEMENTARY Table 4 - Results of answers about open-ended CVD stratified by rank in Part A.](#_w0i9m7kckmaj) 5

[SUPPLEMENTARY Table 5 - Results of answers about open-ended CVD risk factors stratified by rank in Part A.](#_7mqyg0thvskz) 5

[SUPPLEMENTARY Table 6 - Results of Part B: Close-ended knowledge assessment.](#_u86v4hvq5uh) 6

[SUPPLEMENTARY Table 7 - Results of answers about close-ended CVD stratified by rank in Part B.](#_h792miti4eq4) 7

[SUPPLEMENTARY Table 8 - Results of answers about close-ended CVD risk factors stratified by rank in Part B.](#_6t43wk8pn32u) 7

[SUPPLEMENTARY Table 9 - Results of mean combined awareness score for CVD.](#_my092v3kxaa1) 7

[SUPPLEMENTARY Table 10 - Results of mean combined awareness score for CVD risk factors.](#_1cujkogoybf5) 7

[SUPPLEMENTARY Table 11 - Multivariate regression was performed with gender and rank as independent variables.](#_8ydcxsxdwek0) 8

[SUPPLEMENTARY Table 12 - Results of Part A mistakes on open-ended questions on CVDs and CDV risk factors](#_7iefo6ctgxit) **8**

#### SUPPLEMENTARY Table 1 - Demographic and Educational features of the respondents.

| **Demographic feature of the respondents** | **Officers**  **(years)** | **NCO**  **(years)** | **Enlisted**  **(years)** |
| --- | --- | --- | --- |
| Age (minimum) | 18 | 26 | 22 |
| Age (maximum) | 59 | 57 | 55 |
| Age (1st quartile) | 21 | 43 | 33 |
| Age (median) | 23 | 48 | 39 |
| Age (3rd quartile) | 33 | 51 | 42 |
| **Educational feature of the respondents** | **Officers n°**  **(tot = 517)** | **NCO n°**  **(tot = 110)** | **Enlisted n°**  **(tot = 158)** |
| Education (Middle School Diploma) | 1 | 7 | 20 |
| Education (High School Diploma) | 330 | 75 | 130 |
| Education (Bachelor’s Degree) | 113 | 28 | 8 |
| Education (Master’s Degree) | 73 | 0 | 0 |

#### SUPPLEMENTARY Table 2 - Answers of CVD reported in Part A.

| **CVD reported in Part A** | **Officers n° (tot = 517)** | **Officers %** | **NCO n°**  **(tot = 110)** | **NCO %** | **Enlisted n° (tot = 158)** | **Enlisted %** |
| --- | --- | --- | --- | --- | --- | --- |
| Acute Myocardial Infarction | 262 | 50,7 | 61 | 55,5 | 65 | 41,1 |
| Arterial Hypertension | 187 | 36,2 | 50 | 45,5 | 44 | 27,8 |
| Stroke | 121 | 23,4 | 17 | 15,5 | 41 | 25,9 |
| Thrombosis | 75 | 14,5 | 7 | 6,4 | 8 | 5,1 |
| Heart Failure | 63 | 12,2 | 12 | 10,9 | 11 | 7,0 |
| Arrhythmias | 60 | 11,6 | 10 | 9,1 | 14 | 8,9 |
| Atherosclerosis | 26 | 5,0 | 2 | 1,8 | 2 | 1,3 |
| Valvular Heart Disease | 23 | 4,4 | 2 | 1,8 | 1 | 0,6 |
| Myocarditis | 15 | 2,9 | 2 | 1,8 | 3 | 1,9 |
| Cardiomyopathy | 12 | 2,3 | 1 | 0,9 | 2 | 1,3 |
| Aneurysm | 11 | 2,1 | 2 | 1,8 | 2 | 1,3 |
| Pulmonary Embolism | 11 | 2,1 | 0 | 0,0 | 0 | 0,0 |
| Pericarditis | 7 | 1,4 | 0 | 0,0 | 2 | 1,3 |
| Cronic Kidney Disease | 5 | 1,0 | 0 | 0,0 | 0 | 0,0 |
| Peripheral Artery Disease | 5 | 1,0 | 3 | 2,7 | 3 | 1,9 |
| Cardiorespiratory Arrest | 3 | 0,6 | 0 | 0,0 | 0 | 0,0 |
| Aortic Dissection | 2 | 0,4 | 0 | 0,0 | 0 | 0,0 |
| Congenital Malformations | 2 | 0,4 | 1 | 0,9 | 0 | 0,0 |
| Patent Foramen Ovale | 1 | 0,2 | 0 | 0,0 | 0 | 0,0 |
| SyPOpe | 0 | 0,0 | 0 | 0,0 | 1 | 0,6 |

#### SUPPLEMENTARY Table 3 - Answers of CVD risk factors reported in Part A.

| **CVD risk factors reported in Part A** | **Officers n°**  **(tot = 517)** | **Officers %** | **NCO n°**  **(tot = 110)** | **NCO %** | **Enlisted n°**  **(tot = 158)** | **Enlisted %** |
| --- | --- | --- | --- | --- | --- | --- |
| Smoke | 298 | 57,6 | 73 | 66,4 | 91 | 57,6 |
| Sedentary Lifestyle | 196 | 37,9 | 50 | 45,5 | 57 | 36,1 |
| Obesity | 177 | 34,2 | 36 | 32,7 | 41 | 25,9 |
| Diet | 167 | 32,3 | 53 | 48,2 | 61 | 38,6 |
| Dyslipidemia | 115 | 22,2 | 22 | 20,0 | 24 | 15,2 |
| Alcohol | 96 | 18,6 | 33 | 30,0 | 40 | 25,3 |
| Arterial Hypertension | 75 | 14,5 | 17 | 15,5 | 20 | 12,7 |
| Diabetes Mellitus | 75 | 14,5 | 8 | 7,3 | 14 | 8,9 |
| Familiarity | 61 | 11,8 | 13 | 11,8 | 9 | 5,7 |
| Stress | 44 | 8,5 | 17 | 15,5 | 12 | 7,6 |
| Mistakes | 36 | 7,0 | 7 | 6,4 | 6 | 3,8 |
| Age | 25 | 4,8 | 0 | 0,0 | 2 | 1,3 |
| Male | 9 | 1,7 | 0 | 0,0 | 1 | 0,6 |
| Red Meat | 5 | 1,0 | 1 | 0,9 | 1 | 0,6 |
| Salt-Rich Diet | 4 | 0,8 | 1 | 0,9 | 2 | 1,3 |
| Atherosclerosis | 3 | 0,6 | 0 | 0,0 | 0 | 0,0 |
| COVID | 2 | 0,4 | 0 | 0,0 | 0 | 0,0 |
| Menopause | 1 | 0,2 | 0 | 0,0 | 0 | 0,0 |

#### SUPPLEMENTARY Table 4 - Results of answers about open-ended CVD stratified by rank in Part A.

| **Rank** | **open-ended CVD mean score** | **open-ended CVD score SD** | **open-ended CVD median score** |
| --- | --- | --- | --- |
| Officer | 1.723 | 1.581 | 2 |
| PO | 1.545 | 1.097 | 2 |
| Enlisted | 1.259 | 1.232 | 1 |

#### SUPPLEMENTARY Table 5 - Results of answers about open-ended CVD risk factors stratified by rank in Part A.

| **Rank** | **open ended CVD risk factors mean score** | **open ended CVD risk factors score SD** | **open ended CVD risk factors median score** |
| --- | --- | --- | --- |
| Officer | 2.617 | 1.932 | 3 |
| PO | 2.945 | 1.387 | 3 |
| Enlisted | 2.373 | 1.706 | 3 |

#### SUPPLEMENTARY Table 6 - Results of Part B: Close-ended knowledge assessment.

| **Percentage of correct answers regarding CVD awareness from Closed-Ended questionnaire** | | | |
| --- | --- | --- | --- |
| **Rank** | **Enlisted** | **NCO** | **Officer** |
| Arterial Hypertension | 78.48 | 88.18 | 87.81 |
| Acute Myocardial Infarction | 78.48 | 85.45 | 82.94 |
| Heart Failure | 74.05 | 89.09 | 82.01 |
| Cholesterol | 17.72 | 27.27 | 29.91 |
| Diabetes Mellitus | 41.77 | 44.54 | 48.16 |
| Hepatic Steatosis | 49.37 | 47.27 | 60.54 |
| Alzheimer | 64.55 | 58.18 | 80.66 |
| **Percentage of correct answers regarding CVD risk factors awareness from Closed-Ended questionnaire** | | | |
| Smoke | 93.67 | 98.18 | 87.81 |
| Arterial Hypertension | 85.44 | 95.45 | 85.10 |
| LDL<70 | 29.74 | 29.74 | 44.76 |
| LDL>100 | 59.49 | 73.63 | 71.76 |
| Hepatic Steatosis | 29.74 | 35.45 | 38.87 |
| Pneumonia | 56.32 | 58.18 | 65.57 |
| Elevated Transaminases | 20.88 | 31.81 | 22.05 |
| Red Meat | 37.97 | 49.09 | 46.03 |
| Sedentary Lifestyle | 77.84 | 86.36 | 87.62 |
| Obesity | 93.03 | 94.54 | 90.71 |
| Vegan Diet | 58.86 | 60.00 | 64.41 |
| Diabetes Mellitus | 18.98 | 16.36 | 33.84 |
| threshold values of total cholesterol | 48.10 | 62.72 | 59.76 |
| threshold values of arterial pressure | 8.86 | 16.36 | 17.40 |

#### SUPPLEMENTARY Table 7 - Results of answers about close-ended CVD stratified by rank in Part B.

| Rank | close-ended CVD mean score | close-ended CVD score SD | close-ended CVD median score |
| --- | --- | --- | --- |
| Officer | 4.726 | 1.719 | 5 |
| PO | 4.4 | 1.778 | 4 |
| Enlisted | 4.044 | 1.913 | 4 |

#### SUPPLEMENTARY Table 8 - Results of answers about close-ended CVD risk factors stratified by rank in Part B.

| **Rank** | **close ended CVD RF mean score** | **close ended CVD RF score SD** | **close ended CVD RF median score** |
| --- | --- | --- | --- |
| Officer | 6.157 | 2.695 | 8 |
| PO | 8.109 | 2.219 | 8 |
| Enlisted | 7.19 | 2.704 | 7 |

#### SUPPLEMENTARY Table 9 - Results of mean combined awareness score for CVD.

| **Rank** | **combined CVD mean score** | **combined CVD score SD** | **combined CVD median score** |
| --- | --- | --- | --- |
| Officer | 6.422 | 2.720 | 7 |
| PO | 5.945 | 2.270 | 6 |
| Enlisted | 5.304 | 2.541 | 5 |

#### SUPPLEMENTARY Table 10 - Results of mean combined awareness score for CVD risk factors.

| **Rank** | **combined CVD RF mean score** | **combined CVD RF score SD** | **combined CVD RF median score** |
| --- | --- | --- | --- |
| Officer | 10.760 | 3.855 | 11 |
| PO | 11.050 | 2.815 | 11 |
| Enlisted | 9.563 | 3.559 | 10 |

#### SUPPLEMENTARY Table 11 - Multivariate regression was performed with gender and rank as independent variables.

|  | **Dependent variable** | |
| --- | --- | --- |
| **Independent variables** | combined CVD awareness  (p-value) | combined CVD risk factors awareness (p-value) |
| Gender (Male) | -0.597 * (0.03) | -0.976 * (0.1) |
| Rank (PO) | 0.670 * (0.04) | 1.538 *** (0.0007) |
| Rank (Officer) | 1.033 *** (<0.0001) | 1.056 ** (0.002) |
| combined CVD awareness: F-statistic: 9.144 on 3 and 781 DF, p-value: 5.952e-06 | | |
| combined CVD risk factors awareness: F-statistic: 7.283 on 3 and 781 DF, p-value: 8.044e-05 | | |
| Signif. codes: 0 ‘***’ 0.001 ‘**’ 0.01 ‘*’ 0.05 | | |

#### SUPPLEMENTARY Table 12 - Results of Part A mistakes on open-ended questions on CVDs and CDV risk factors

| **Part A mistakes in CVDs knowledge** | | | | | |
| --- | --- | --- | --- | --- | --- |
|  | 0 mistakes (n°, %) | 1 mistakes (n°, %) | 2 mistakes (n°, %) | 3 mistakes (n°, %) | 4 mistakes (n°, %) |
| **Officers**  **(tot = 517)** | 390 (74.4%) | 84 (16.2%) | 31 (6.0%) | 11 (2.1%) | 1 (0.2%) |
| **NCO**  **(tot = 110)** | 90 (81.8%) | 15 (13.6%) | 3 (2.7%) | 1 (0.9%) | 1 (0.9%) |
| **Enlisted**  **(tot = 158)** | 124 (78.5%) | 28 (17.7%) | 4 (2.5%) | 2 (1.3%) | 0 (0%) |
| **Part A mistakes in CVD risk factors knowledge** | | | | | |
|  | 0 mistakes (n°, %) | 1 mistakes (n°, %) | 2 mistakes (n°, %) | 3 mistakes (n°, %) | 4 mistakes (n°, %) |
| **Officers**  **(tot = 517)** | 476 (92.1%) | 36 (7.0%) | 5 (0.9%) | 0 (0%) | 0 (0%) |
| **NCO**  **(tot = 110)** | 102 (92.7%) | 7 (6.4%) | 1 (0.9%) | 0 (0%) | 0 (0%) |
| **Enlisted**  **(tot = 158)** | 152 (96.2%) | 6 (3.8%) | 0 (0%) | 0 (0%) | 0 (0%) |
